# The ancient viral whispers of ageing: awakening the dark genome

**DOI:** 10.1093/lifemedi/lnag023

**Published:** 2026-06-24

**Authors:** Hui Zhang, Guang-Hui Liu, Jing Qu

**Affiliations:** Beijing Institute of Heart Lung and Blood Vessel Diseases, Beijing Anzhen Hospital, Capital Medical University, Beijing 100029, China; State Key Laboratory of Organ Regeneration and Reconstruction, Human Organ Physiopathology Emulation System, Institute of Zoology, Chinese Academy of Sciences, Beijing 100101, China; University of Chinese Academy of Sciences, Beijing 100049, China; Beijing Institute for Stem Cell and Regenerative Medicine, Beijing 100101, China; Aging Biomarker Consortium (ABC), Beijing 100101, China; Beijing Institute of Heart Lung and Blood Vessel Diseases, Beijing Anzhen Hospital, Capital Medical University, Beijing 100029, China; State Key Laboratory of Organ Regeneration and Reconstruction, Human Organ Physiopathology Emulation System, Institute of Zoology, Chinese Academy of Sciences, Beijing 100101, China; University of Chinese Academy of Sciences, Beijing 100049, China; Beijing Institute for Stem Cell and Regenerative Medicine, Beijing 100101, China; Aging Biomarker Consortium (ABC), Beijing 100101, China

For decades, the about 8% of the human genome that traces back to ancient retroviral infections was dismissed as ‘junk DNA’—the fossilized remnants of evolutionary battles long won. However, a growing body of recent work has resurrected a startling idea: these endogenous retroviruses (ERVs) are not merely silent passengers. As we age, they awaken. Importantly, accumulating evidence suggests that their reawakening actively drives the ageing process itself, as well as age-related diseases of the brain, the heart and beyond.

The first key insight came from senescent human mesenchymal progenitor cells, where the most recently integrated human ERV—HERVK (HML-2)—becomes unlocked from its epigenetic cage. Hypomethylation and loss of the repressive histone mark H3K9me3 allow HERVK to transcribe viral genes and even assemble into retrovirus-like particles (RVLPs). Strikingly, these RVLPs are released and taken up by young, healthy cells, where they trigger senescence in a contagious, paracrine manner via the cytosolic DNA sensor cGAS and the STING pathway, unleashing a type I interferon response and a cascade of inflammatory senescence-associated secretory phenotype (SASP) factors [1] (Fig. 1). This phenomenon is not restricted to dividing cells. In post-mitotic neurons of the ageing primate frontal lobe—a region exquisitely vulnerable to cognitive decline—erosion of the nuclear lamina and loss of B-type lamins lead to heterochromatin disorganization and consequent ERV derepression, again activating cGAS–STING signalling and fuelling neuroinflammation [2] (Fig. 1). Together, these findings indicate that the ERV-driven cascade operates across both mitotic and post-mitotic cells, suggesting it may be a widespread feature of mammalian ageing [3]. Nevertheless, definitive genetic evidence that ERV derepression acts as an initiating cause rather than a propagating amplifier of organismal ageing is still lacking.

**Figure 1.**
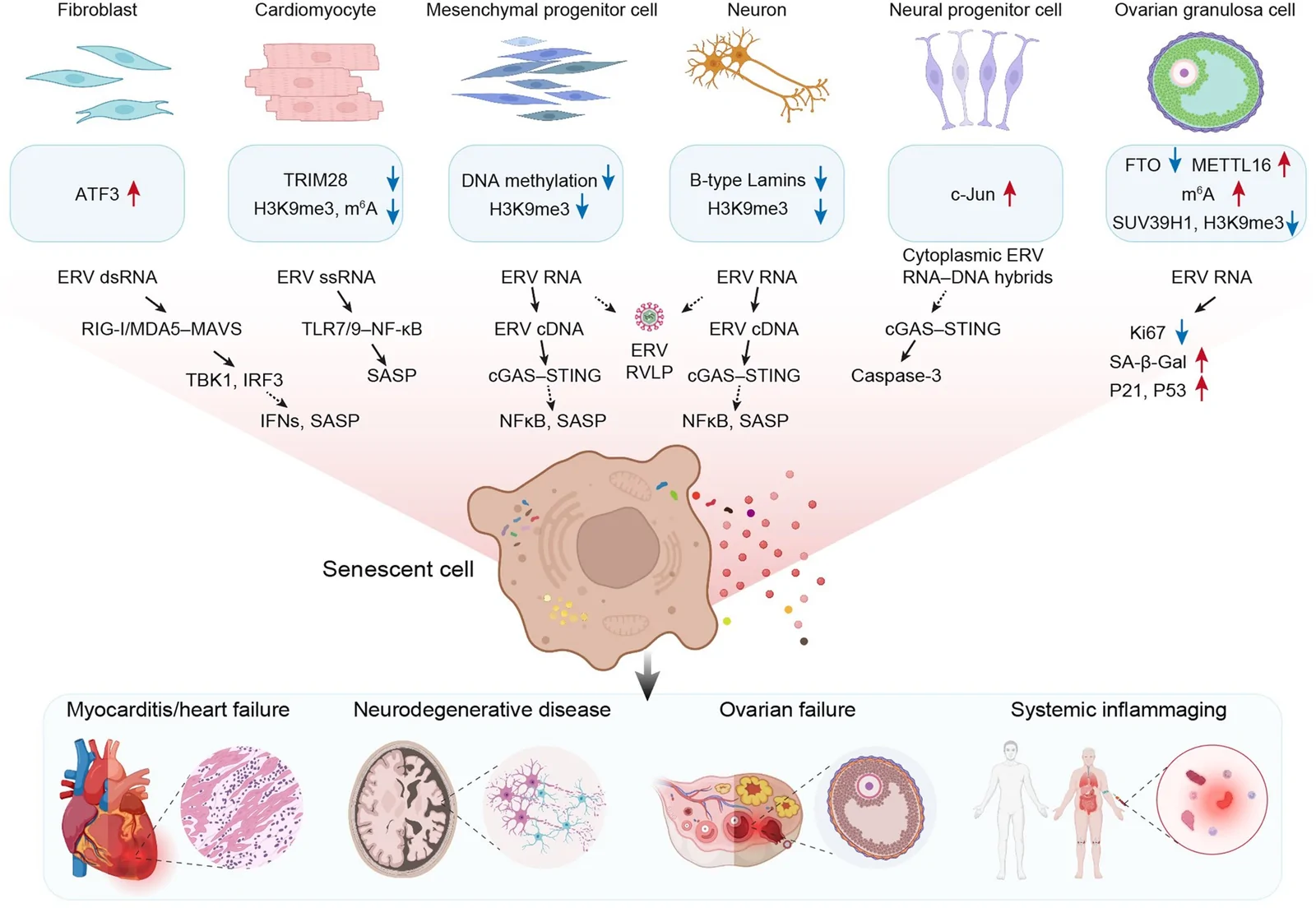
Reactivation of ERVs drives ageing and age-related pathologies across multiple tissues and cell types.Ageing induces progressive epigenetic erosion in diverse somatic cells, which relieves the epigenetic silencing of ERVs embedded in the human genome. The derepressed ERVs are transcribed and produce multiple nucleic acid products including RVLPs, double-stranded RNA (dsRNA), single-stranded RNA (ssRNA), RNA–DNA hybrids and ERV-derived complementary DNA (cDNA) in a cell-type-dependent manner. These diverse ERV products are recognized by distinct pattern recognition receptors of the innate immune system, primarily three core signalling axes: the cGAS–STING pathway, the RIG-I/MDA5–MAVS pathway, and the TLR7/9–NF-κB pathway. All these immune cascades converge to trigger chronic sterile inflammation, cellular senescence, and the secretion of SASP factors, ultimately leading to tissue-specific ageing phenotypes and age-associated diseases. Specifically, in human mesenchymal progenitor cells, loss of DNA methylation and repressive histone mark H3K9me3 unlock ERVs to generate RVLPs, which propagate senescence to neighbouring cells via the cGAS–STING pathway [1]. In ageing postmitotic neurons of the primate prefrontal lobe, erosion of nuclear lamina and depletion of B-type lamins disrupt heterochromatin structure and reactivate ERVs, subsequently activating cGAS–STING signalling and neuroinflammation linked to neurodegenerative disorders [2]. In senescent fibroblasts, the age-dependent accessibility of transcription factor ATF3 drives bidirectional transcription of ERVs to generate dsRNA, which activates the RIG-I/MDA5–MAVS pathway to initiate interferon responses and SASP [4]. In cardiomyocytes during heart failure, ERV reactivation predominantly stimulates the TLR7/9-NF-κB cascade, resulting in myocarditis and acute heart failure [6]. In hippocampal neural progenitors associated with Alzheimer’s disease, upregulated c-Jun promotes HERVK activation and cytoplasmic RNA–DNA hybrid accumulation, eliciting cGAS–STING-mediated cell death [7]. In ovarian granulosa cells, altered m^6^A RNA methylation (caused by FTO downregulation and METTL16 upregulation) reduces SUV39H1 and H3K9me3 levels, de-represses ERV1, and activates autophagy and apoptosis, eventually causing ovarian follicle loss and ovarian failure [9]. Collectively, ERV reactivation acts as a conserved upstream driver of systemic inflammaging, cellular senescence, and a spectrum of tissue-specific age-related disorders across mitotic and postmitotic cells. Ki67 and SA-β-Gal serve as classical biomarkers for proliferative activity and cellular senescence, respectively; P21, P53 and Caspase-3 are key effector molecules mediating cell cycle arrest and cell death downstream of ERV activation. This figure was generated using BioRender.

The pathways of ERV reactivation are not monolithic. In senescent human fibroblasts, the transcription factor ATF3—whose binding motif becomes accessible with age—directly activates a specific subclass of ERVs, driving bidirectional transcription that produces double-stranded RNA (dsRNA). This dsRNA is sensed by the RIG-I/MDA5–MAVS pathway, again triggering an interferon response and SASP [4] (Fig. 1). ERVs thus engage both DNA and RNA sensing arms of the innate immune system, depending on cell type and the nature of the ERV transcript. Furthermore, RNA–DNA hybrids derived from ERV transcripts have been identified as unexpected effectors in the prefrontal cortex in models of autism spectrum disorder, where they drive expression of the complement protein C4b and instruct microglia to excessively prune synapses—a phenotype rescued by reverse transcriptase inhibitors [5]. In the failing heart, ERV activation in cardiomyocytes triggers the TLR7/9-NF-κB pathway, leading to myocarditis and acute heart failure [6] (Fig. 1). In Alzheimer’s disease, c-Jun upregulation drives HERVK reactivation, cytoplasmic RNA–DNA hybrid accumulation and cGAS–STING-mediated cell death in hippocampal progenitors [7] (Fig. 1). Collectively, these findings reveal that ERVs can mobilize multiple innate immune sensors—cGAS–STING, RIG-I–MAVS and TLR7/9—all converging on chronic, sterile inflammation. An unresolved question is whether the choice of sensor is dictated by the class of ERV derepressed, the cell-type-specific expression of pattern recognition receptors, or the kinetic phase of the senescence program.

The clinical translation of these insights is accelerating. An epigenetic clock based solely on DNA methylation states of HERVs and LINEs—termed Retroelement Age—predicts chronological age with high accuracy, is accelerated in HIV infection, reversed by antiretroviral therapy, and responds to Yamanaka factor reprogramming, offering a new quantitative biomarker to monitor ageing interventions [8]. In the ovary, increased m^6^A RNA methylation (due to FTO loss and METTL16 gain) downregulates SUV39H1 and H3K9me3, derepressing ERV1 and activating autophagy and apoptosis, leading to follicle loss—revealing an epitranscriptomic layer of control over ERV silencing [9] (Fig. 1). Most importantly from a therapeutic standpoint, abacavir and other FDA-approved reverse transcriptase inhibitors, originally developed for HIV, have shown efficacy in preclinical models of osteoarthritis, brain ageing, autism and heart failure. Nevertheless, several challenges remain. First, the efficacy of these inhibitors against endogenous retrotransposon activity in human ageing contexts requires further validation at clinically relevant concentrations. Second, chronic inhibition of endogenous reverse transcriptase may carry unforeseen risks, given emerging evidence for beneficial roles of ERV-derived elements in development and neural plasticity. The ancient viral whispers in our genome are no longer noise—they are a potentially targetable vulnerability, a biomarker of biological age [10], and perhaps a lever by which to modulate the volume of ageing itself.

## References

[1] Liu X , Liu Z, Wu Z et al Resurrection of endogenous retroviruses during aging reinforces senescence. Cell 2023;186:287–304.e226. 10.1016/j.cell.2022.12.017 36610399

[2] Zhang H , Li J, Yu Y et al Nuclear lamina erosion-induced resurrection of endogenous retroviruses underlies neuronal aging. Cell Rep 2023;42:112593. 10.1016/j.celrep.2023.112593 37261950

[3] Zeng Q , Wang W, Tian W et al Cell-type-specific transposon demethylation and TAD remodeling in aging mouse brain. Cell 2026;189:2148–66.e2127. 10.1016/j.cell.2026.02.015 PMC13007725 41819104

[4] Mao J , Zhang Q, Zhuang Y et al Reactivation of senescence-associated endogenous retroviruses by ATF3 drives interferon signaling in aging. Nat Aging 2024;4:1794–812. 10.1038/s43587-024-00745-6 39543280

[5] Chen S , Zhang B, Qin T et al Endogenous retrovirus-derived RNA-DNA hybrids induce microglial synaptic pruning in autism models. Neuron 2026;114:1765–81.e7. 10.1016/j.neuron.2026.01.011 41742408

[6] Xiong J , Zhang S, Geng Z et al An aberrant resurgence of endogenous retroviruses prompts myocarditis and heart failure. Circulation 2025;152:939–56. 10.1161/CIRCULATIONAHA.125.074845 PMC12466171 40820798

[7] Scopa C , Barnada SM, Cicardi ME et al JUN upregulation drives aberrant transposable element mobilization, associated innate immune response, and impaired neurogenesis in Alzheimer’s disease. Nat Commun 2023;14:8021. 10.1038/s41467-023-43728-8 PMC10696058 38049398

[8] Ndhlovu LC , Bendall ML, Dwaraka V et al Retro-age: a unique epigenetic biomarker of aging captured by DNA methylation states of retroelements. Aging Cell 2024;23:e14288. 10.1111/acel.14288 PMC11464121 39092674

[9] Hu X , Lu J, Ding C et al The N6-methyladenosine landscape of ovarian development and aging highlights the regulation by RNA stability and chromatin state. Aging Cell 2025;24:e14376. 10.1111/acel.14376 PMC11822672 39410722

[10] Wu Z , Ma S, Wang S et al The entropic view of aging: from thermodynamics to biology. Life Medicine 2026;5:lnag020. 10.1093/lifemedi/lnag020 PMC13319325 42388853

